# Limitations of the use of the Glasgow Coma Scale in intensive care patients with non-neurological primary disease: a search for alternatives

**DOI:** 10.1186/cc9926

**Published:** 2011-03-11

**Authors:** PV Dong, OL Cremer

**Affiliations:** 1 University Medical Centre Utrecht, the Netherlands

## Introduction

Numerous scoring systems have been devised to assess the severity of illness and predict outcome in critically ill patients in the ICU, many of which incorporate the Glasgow Coma Scale (GCS) as a key component. However, the GCS requires observation of a verbal score (which is often unavailable in the ICU), must be interpreted in cases of concurrent sedation, and is insensitive to more subtle derangements of consciousness (such as delirium). Furthermore, its relationship with outcome may be nonlinear. In this study we quantified the practical limitations of using the GCS in daily routine. We then aimed to provide alternative methods for neurological assessment scoring in case of missing GCS scores.

## Methods

We performed an observational study of all patients admitted to a large tertiary ICU from January 2009 until September 2010. Patients following elective surgery, having an uncomplicated stay <96 hours, were excluded from analysis. We collected data on neurological status and sedation. All variables were assessed for their ability to predict hospital mortality, using multivariate logistic regression analyses that included the variables of primary interest as well as any relevant covariates.

## Results

In total 1,128 patients were included (62% males, mean age 58 ± 17 years, 40% surgical admissions). We observed an overall 26% hospital mortality rate (compared with 30% predicted by the APACHE IV model). In patients with maximum GCS motor scores of M1 and M2-3 on their first day in the ICU, the mortality rate was 62% and 79%, respectively. Within the large majority of patients with a M6 score, we observed a broad range of clinical variance, expressing low discriminative ability of the GCS motor score. We found inferior predictive power of the APACHE IV model in patients with non-neurological primary disease (*c *statistic = 0.75 to 0.79) compared with patients with acute neurological injury (0.85 to 0.86). The predictive power of the APACHE IV model improved when substituting missing GCS components by other neurological observables.

## Conclusions

The GCS is difficult to obtain and interpret, and shows inconsistent predictive power. In patients with non-neurological primary disease, the use of alternative observables, such as pupillary anomaly, RASS score and sedative use, may serve as a substitute score in cases of missing or unobservable GCS assessments.
